# Selection and Validation of Reference Genes for Gene Expression Studies in *Codonopsis pilosula* Based on Transcriptome Sequence Data

**DOI:** 10.1038/s41598-020-58328-5

**Published:** 2020-01-28

**Authors:** Lijun Liang, Zhigui He, Haizheng Yu, Erhuan Wang, Xiaojiang Zhang, Bingxue Zhang, Chenlu Zhang, Zongsuo Liang

**Affiliations:** 1 Institute of soil and water conservation, CAS & MWR, Yangling, 712100 P. R. China; 2 College of Landscape Architecture, Zhejiang Agriculture & Forestry University, Hangzhou, 311300 P. R. China; 3 University of Chinese Academy of Sciences, Beijing, 100049 P. R. China; 4 College of Life Sciences, Northwest A&F University, Yangling, 712100 P. R. China; 5 Institute of Food Science and Biological Engineering, Guilin Tourism University, Guilin, 541006 P. R. China; 6 Buchang Pharmaceuticals Co., Ltd, Xi’an, 712000 P. R. China; 7 School of Biological Science and Engineering, Shaanxi University of Technology, Hanzhong, 723000 P. R. China; 8 College of Life Sciences, Key Laboratory of Plant Secondary Metabolism and Regulation of Zhejiang Province, Zhejiang Sci-Tech University, Hangzhou, 310018 P. R. China

**Keywords:** Transcriptomics, Plant molecular biology

## Abstract

Relative gene expression analyses by RT-qPCR (reverse transcription-quantitative PCR) are highly dependent on the reference genes in normalizing the expression data of target genes. Therefore, inappropriate endogenous control genes will lead to inaccurate target gene expression profiles, and the selection and validation of suitable internal reference genes becomes essential. In this study, we retrieved the commonly used reference genes in transcriptome datasets of *Codonopsis pilosula* by RNA-Seq (unpublished data), and selected 15 candidate reference genes according to the coefficient of variation (CV) and fold change (FC) value of gene expression. The expression levels of candidate reference genes, which is at different growth stages, undergoing cold stress and drought stress, was determined by RT-qPCR. The expression stability of these genes was evaluated using software packages and algorithms including ΔCt, geNorm, NormFinder and Bestkeeper. Then appropriate reference genes were screened and validated by target gene-*UDGPase* (UDP glucose pyrophosphorylase). The optimal RGs combinations of *C. pilosula*, including *PP2A59γ*, *CPY20-1*, *UBCE32*, *RPL5B* and *UBC18* for developmental stage, *RPL5B*, *RPL13* and *PP2A59γ* for cold treatment, *RPL13* and *PP2A59γ* for drought treatment, were found and proposed as reference genes for future work. This paper laid foundations for both the selection of reference genes and exploration in metabolic mechanism of *C. pilosula*.

## Introduction

*Codonopsis pilosula* (Franch.) Nannf) is a traditional Chinese medicinal and edible plant^1,2^. As a history of more than 300 years, *C. pilosula* is mainly distributed in four provinces of China including Gansu, Shannxi, Shanxi and Hubei^2,3^. It is reported that the main ingredients related to its pharmacodynamics are polysaccharide, tangshenosides, lobetyolin, atractylenolide, and so on^4^. By means of transcriptome sequencing, Gao J. P. *et al*. and Li J. *et al*. revealed that *UDGPase* (UDP glucose pyrophosphorylase) and *UGDH* (UDP-glucose dehydrogenase) were the key enzyme genes in *C. pilosula* controlling the synthesis pathway of polysaccharide^5–8^. Consequently, *UDGPase* was cloned and characterized by them adopting *GAPDH* as reference gene (RG)^8^. *GAPDH* and *SUC4* (sucrose/H^+^ cotransporter 4) were also cloned and characterized by Wang X. L. *et al*. and Zheng Q. H. *et al*.^5,6^. In the above studies, Real-Time Quantitative PCR (RT-qPCR) was commonly used in expression analysis of those genes.

Compared with traditional quantitative analysis techniques like Northern blots or cDNA microarrays, RT-qPCR is much more popular in gene expression because of its time-saving, accuracy, low cost and high throughput characteristics^9–16^. RT-qPCR has played an important role in researching the molecular regulation of secondary metabolites of medicinal plants. Wu Y. N. *et al*. and Gao J. P. *et al*. analyzed several gene expression of *C. pilosula*, which laid foundations for the gene regulation. Quantitative method is crucial to the accuracy of gene expression in RT-qPCR, which is divided into absolute quantification and relative quantification methods^10,17^. The latter is well received for its easy operation characteristics. This method is highly dependent on the constant of internal reference genes (RGs). Accordingly, stable RGs without significant variance in all the tested tissues, during the different period of development, and under different cultivating conditions are required^10–12,14,15,18–20^. RGs, which expressed in all cells of plants as housekeeping genes, mainly include *ACT* (actin), *GAPDH* (glyceraldehyde-3-phosphate dehydrogenase), *G6PDH* (glucose-6-phosphate dehydrogenase), *EF* (elongation factor), *18* *S rRNA* (18 S ribosomal RNA), *UBQ* (ubiquitin), *TUB* (tubulin), *EIF* (eukaryocyte initiation factor), *UBC* (ubiquitin conjugating enzyme), *H3* (histone), *PGK* (phosphoglycerate kinase), *TEF* (translation elongation factor), *CYP* (Cyclophilin), *NADHD* (Nicotinamide adenine dinucleotide dehydrogenase), and so on^9,19,21–27^. Recent studies have shown that gene expression was highly tissue-specific and often varied with the physiological status of the plant or experimental conditions, and the universal reference genes have not been found at present^19,21,24,28–30^. Selection of RGs should be conducted amongst the same pool of samples, and carried out according to the specific tissue, developmental stages and experimental conditions, thus the selected RGs can be used for target gene expression^25,31–35^. In Gao J. P.’s study on the polysaccharide of *C. pilosula*, *GAPDH* was used as the internal reference gene to normalize the expression of *UDGPase*. This work started the exploration of synthesis mechanism for *C. pilosula* metabolites, and *GAPDH* became the first internal reference gene used in the study of molecular regulation mechanisms. In order to make clear the mechanisms, systematic selection and validation of RGs are required. Reliable internal reference genes were particularly essential for *C. pilosula* during the growth stages and above all under the drought and cold stress conditions, because *C. pilosula* is manly distributed in dry and cold areas with frequently extreme weather. The impact of extreme climates such as cold and drought on the reliable of RG was extensively reported in many plants other than *C. pilosula*^21,24,31,32^.

In this study, we retrieved the commonly used RGs of *C. pilosula* in transcriptome datasets (unpublished data), and selected 15 candidate RGs according to threshold method of coefficient of variation (CV) and fold change (FC) value in gene expression^36,37^. RT-qPCR was then adopted to determine the candidate RGs at developmental stages, under cold stress treatments and drought stress treatments. The corresponding stability was evaluated using software packages and algorithms including ΔCt^38^, geNorm^39^, NormFinder^40^ and Bestkeeper^41^. Based on the above analysis, the appropriate RGs were screened and validated by target gene-*UDGPase* (UDP glucose pyrophosphorylase). This research for the first time systematically explored the RGs of *C. pilosula* to lay foundations for the selection of RGs and analysis of gene expression.

## Results

### Selection of candidate reference genes based on transcriptome data in *C. pilosula*

Root of *C. pilosula* at the initial and late stages of its swelling were collected, and three tissue layers including periderm, phloem and xylem of the roots were separated as samples for transcriptome sequencing. Six replicates for each tissue. The samples were sent to Lianchuan biological co. LTD to perform the transcriptome sequencing by using paired-end sequencing technology on an Illumina Hi-Seq™ 4000 platform. After assembling and annotation, expression profile data was mapped to the transcriptome. TPM (Transcripts per kilobase of exon model per million mapped reads) was calculated by means of EdgeR package (The RSEM package), which was then used to analyze gene expression level.

According to literatures on the conventional RGs, the related genes including *ACT*, *GAPDH*, *G6PDH*, *EF*, *18* *S rRNA*, *UBQ*, *TUB*, *EIF*, *UBC*, *H3*, *PGK*, *TEF*, *CYP* and *NADHD* were retrieved from the transcriptomic data of *C. pilosula*. As is reported, low-expression genes would make poor RT-qPCR references due to the difficulties in detecting and quantifying their expression, thus, genes with expression levels lower than five TPM were excluded before the stability analyses. In addition, genes with log fold variance more than two in different tissues were also rejected. After the removals, a total of 207 genes were selected for further study. Calculations for mean value (MV), SD, CV and log_2_FC were executed in Microsoft Excel according to TPM^27^. Genes with low CV and log_2_FC were considered stable. We set 0.5 as the CV cut-off value for stable genes and ranked the selected 207 genes. The top 50 genes were displayed in Table S1. After reducing the cut-off values of CV to 0.3 and |log_2_FC| to 0.2 according to the literatures^11,42–44^, fifteen genes were selected as candidate RGs.

### Expression profile of candidate reference genes of *C. pilosula*

Primers of 15 candidate RGs were used to amplify cDNA template by PCR technology, and single stripe of target amplicon could be obtained, which was consistent with the expected target stripe size. The melting curves of 15 candidate genes’ primers were plotted, all of which showed single peak, indicating that the primer had strong specificity and non-specific amplification occurred. When the length of RT-qPCR amplification product increased from 103 bp to 191 bp, the amplification efficiency of candidate RGs improved from 91.3% to 107.2% (Table 1).

**Table 1:** Primer sequences and amplicon characteristics of 15 candidate RGs and validation gene for RT-qPCR analysis, *gene known to have different levels of expression in *C. pilosula* was used to validate the candidate RGs.

Gene symbol	Gene description	Primer sequences (5′−3′)	Amplicon Size(bp)	TM (°C)	Efficiency (%)
*CYP20-1*	Cytochrome P450 20-1	GGACCAGACACCAATGGTTCAC	188	58.0	91.5
		AAGGGTAGCTCTCCGCTGTC			
*ABCC8*	ABC-transporter C family member 8	AGCCCTAACGGGTACCCAAG	136	58.0	96.2
		TGGCCTCTTGTCCTCCACAA			
*ACTβ*	Beta-Actin	AGAGAAAGCGCTGAAATGCCA	103	58.0	102.1
		AGCCTTGGGACGAAACCCTA			
*UPL-RHF2A*	E3 ubiquitin-protein ligase RHF2A	GAGGGAGGGCTCAAGGAGTC	172	58.0	92.5
		AGCTGCAAATGGGACAGACG			
*EF1α*	Elongation factor 1-alpha	GCCTGGTGACAACGTTGGAT	191	58.0	107.2
		GCGAGGTGTGGCAATCAAGA			
*EIF4A-14*	Eukaryotic initiation factor 4A	GTGGCAGGGAAATCGGTTGT	187	58.0	98.1
		GCTGAATAGCGGAGGGCTTC			
*RPL5B*	60 S ribosomal protein L5	CAGCTGCTTACGCCCATGAA	180	58.0	95
		CCGCCGGTTCAACAGAGAAA			
*RPL13*	60 S ribosomal protein L13	AGGAACTTGCAACAGCCACA	176	58.0	93.7
		CGCAGCCCTCTTAAGCCTTT			
*G6PDH*	glucose-6-phosphate dehydrogenase	TGGTGACGTAGTGCAGAGTC	187	58.0	91.3
		AGCATCAACGTTGTCCTCAGA			
*GAPDH*	Glyceraldehyde 3-phosphate dehydrogenase	CCAAGAAGGCAGGCATGGTT	150	58.0	94.1
		CCTTTACGGCTGTGCACCTT			
*TUBα3*	Alpha-tubulin	TGACTGGTGCCCTACTGGTT	178	58.0	106.4
		CGCCCTCTTTGCGTACATGA			
*PP2A57θ*	Protein phosphatase 2A-57	CCTGCACTGGAGAGAAACGG	183	58.0	102.5
		ACGTTTCCATGCAGCTTCCC			
*PP2A59γ*	Protein phosphatase 2A-59	GCGGACTCACTGGAACCAAG	166	58.0	95.6
		CAGGCGCTTCCAGGTTGATT			
*UBC18*	Ubiquitin-conjugating enzyme 18	CCAGCTCCTCTACACCCTCA	152	58.0	104.7
		TCTTCTGGTCGTTGCTTCGC			
*UBCE32*	Ubiquitin-conjugating enzyme 32	TGGTGACCAGGCAAACGATG	175	58.0	91.8
		CCTCAAGACTGCAGGAGTGG			
UGPase*	UDP-glucose pyrophosphorylase	TGATGGCTATGTGACCCGGAA	187	58.0	102.1
		ACCCTTGAGAACGACGGAAGA			

**Figure 1:**
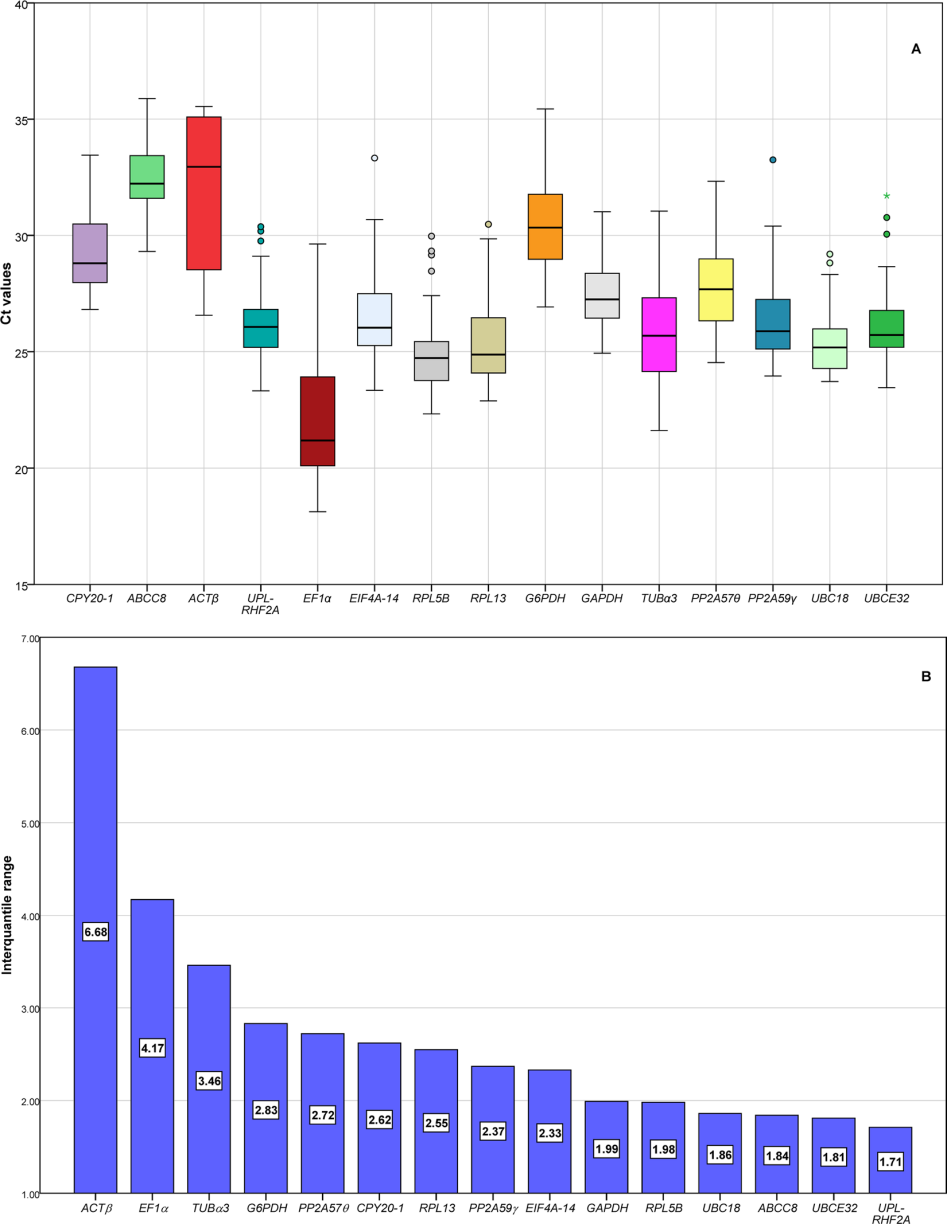
RT-qPCR Ct values and interquartile ranges. (**A**) Ct values for each RG in all samples. A line across the box depicts the median. The box indicates the 25% and 75% percentiles. Whiskers represent the maximum and minimum values, circles represent outliers and asterisks indicate extremes. (**B**) interquartile ranges indicate variability of Ct values among 25% and 75%.

The expression level of candidate RGs was normally determined by the threshold cycle (Ct) values of RT-qPCR. Small Ct value indicated higher gene expression abundance, and vice versa. Figure 1 displayed the Ct values of RGs and their variability. The genes with lower variability were *UPL-RHF2A*, *UBCE32*, *UBC18*, *ABCC8*, and *RPL5B* according to the distribution of raw Ct values, thus could be selected as the RGs. The commonly used *ACTβ*, *EF1α*, and *TUBα3* were the worst candidate RGs because the Ct values spanned multiple units according to the scope of the interquartile. However, analyses on the distribution of the raw Ct values and the range of the interquartile are not sufficient to assess the expression stability of candidate RGs, and other methods are required for more comprehensive and accurate evaluations.

### Expression stability analysis of candidate reference genes of *C. pilosula*

The expression stability of fifteen candidate RGs of leaf, stem, and root of *C. pilosula* under the conditions of different developmental stage, cold stress, drought stress, as well as the total samples were analyzed by means of Δ Ct, geNorm, NormFinder, and BestKeeper methods.

### ΔCt analysis

The mean SD of ΔCt between one gene and the other 14 genes were calculated, which was used to evaluate the stability of the gene. The lower the mean SD was, the higher the stability of the gene tended to. Table 2 listed the rank of RGs and their expression stability values at different developmental stages and treatments. *PP2A59γ* and *RPL5B* were the most stable RGs at the different developmental stages. *RPL5B* was the most stable RG in cold stress treatment, and *PP2A59γ* was most stable in drought stress treatment. *UBCE32* was found to be the most stable RG in the total samples, while *ACTβ* was the most unstable RG both at the different developmental stages and under the cold stress or drought stress treatments.

**Table 2:** The rank of RGs for normalization and their expression stability values calculated by the ΔCt method.

Rank position	Developmental stage		Cold		Drought		Total	
	Gene	Average of STDEV	Gene	Average of STDEV	Gene	Average of STDEV	Gene	Average of STDEV
1	*PP2A59γ*	1.354	*RPL5B*	1.015	*PP2A59γ*	0.812	*UBCE32*	1.185
2	*RPL5B*	1.354	*PP2A59γ*	1.024	*CPY20-1*	0.857	*RPL5B*	1.223
3	*UBCE32*	1.357	*UBCE32*	1.055	*UBCE32*	0.858	*PP2A59γ*	1.253
4	*UBC18*	1.426	*RPL13*	1.064	*RPL13*	0.883	*RPL13*	1.254
5	*G6PDH*	1.446	*EIF4A-14*	1.081	*UBC18*	0.928	*EIF4A-14*	1.282
6	*CPY20-1*	1.464	*PP2A57θ*	1.129	*UPL-RHF2A*	0.931	*CPY20-1*	1.298
7	*EIF4A-14*	1.495	*G6PDH*	1.172	*RPL5B*	0.978	*PP2A57θ*	1.308
8	*PP2A57θ*	1.499	*CPY20-1*	1.177	*PP2A57θ*	0.990	*G6PDH*	1.353
9	*RPL13*	1.523	*EF1α*	1.294	*EIF4A-14*	1.020	*UBC18*	1.386
10	*TUBα3*	1.570	*UPL-RHF2A*	1.299	*ABCC8*	1.060	*TUBα3*	1.464
11	*GAPDH*	1.658	*UBC18*	1.379	*EF1α*	1.069	*UPL-RHF2A*	1.549
12	*ABCC8*	1.851	*TUBα3*	1.379	*G6PDH*	1.206	*GAPDH*	1.579
13	*UPL-RHF2A*	2.077	*GAPDH*	1.519	*TUBα3*	1.221	*ABCC8*	1.692
14	*EF1α*	2.401	*ABCC8*	1.815	*GAPDH*	1.364	*EF1α*	2.179
15	*ACTβ*	2.429	*ACTβ*	2.977	*ACTβ*	2.864	*ACTβ*	2.996

### geNorm analysis

**Figure 2:**
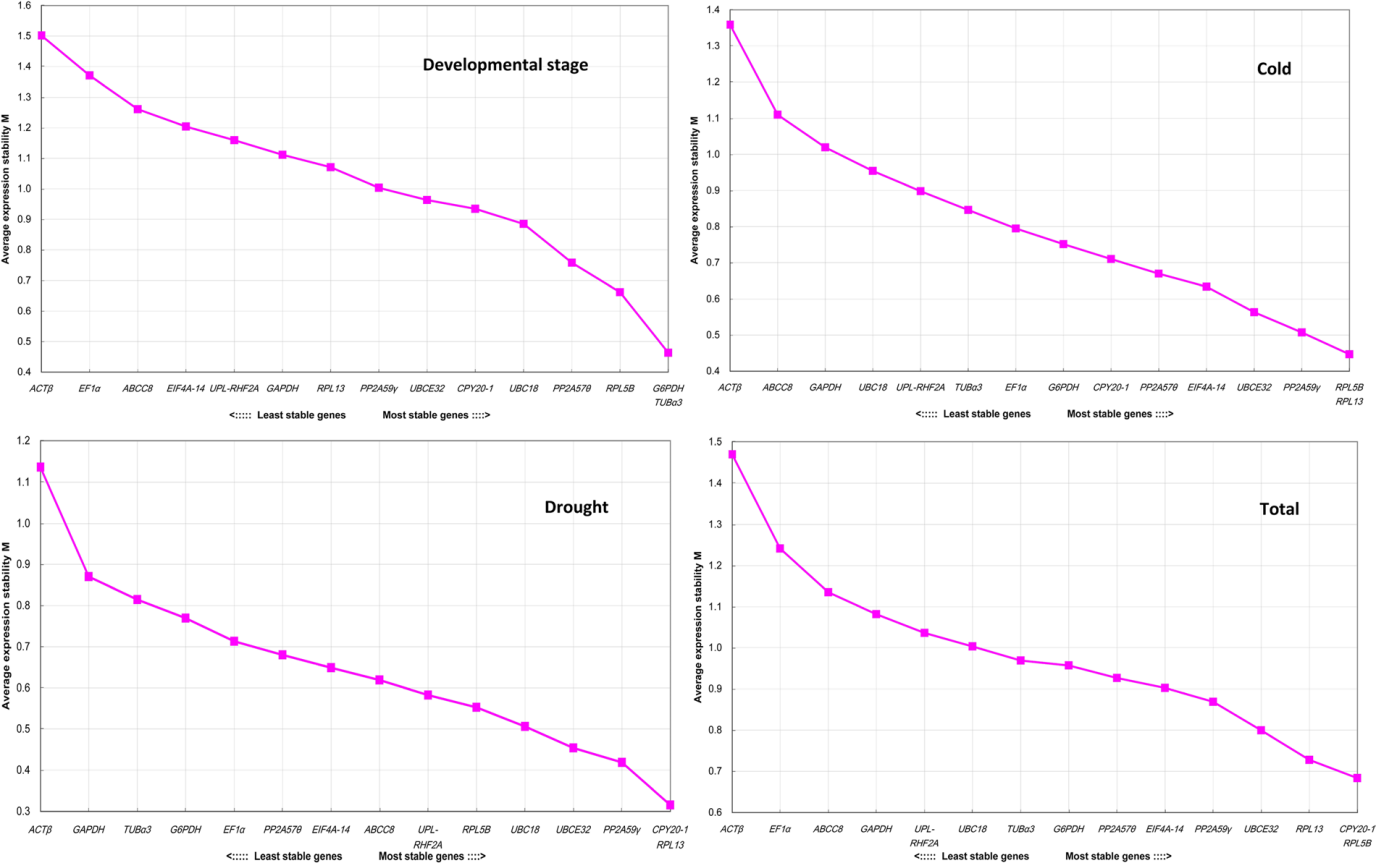
Average expression stability values (M) and ranking of the candidate RGs calculated using geNorm. A lower value of the average expression stability indicates more stable expression.

The M values of fifteen candidate RGs were calculated by using geNorm program and the stability of each candidate RG was ranked by the M value. Genes with the lowest M values are considered to have the most stable expression and denoted as the suitable RG. The samples were divided into four groups: developmental stage (ck, 15d and 30d), cold stress (ck, 4 h, 8 h and 12 h), drought stress (ck, 5d and 10d), and total (all the samples). The average expression stability values (M) and ranking of the candidate RGs was shown in Fig. 2. The optimal RGs were found to vary with the experimental conditions. *CPY20-1* and *UBC18* were the most credible RGs for samples collected at different developmental stages. *RPL5B* and *RPL13* were the best RGs for samples experiencing cold stress treatment. In drought stress treatment, *CPY20-1* and *RPL13* were the most suitable RGs. *CPY20-1* and *RPL5B* were the most stable RGs for all the samples.

To determine the optimal number of RGs for normalization of gene expression level, the pairwise variation (V_n/n+1_) of two sequential normalization factors (NFs) was computed using geNorm program. If the pairwise variation between genes is larger than or equal to 0.15, one gene should be added for reliable normalization of the pairwise variation. Once the pairwise variation is below 0.15, no additional genes are required for normalization^42^.

**Figure 3:**
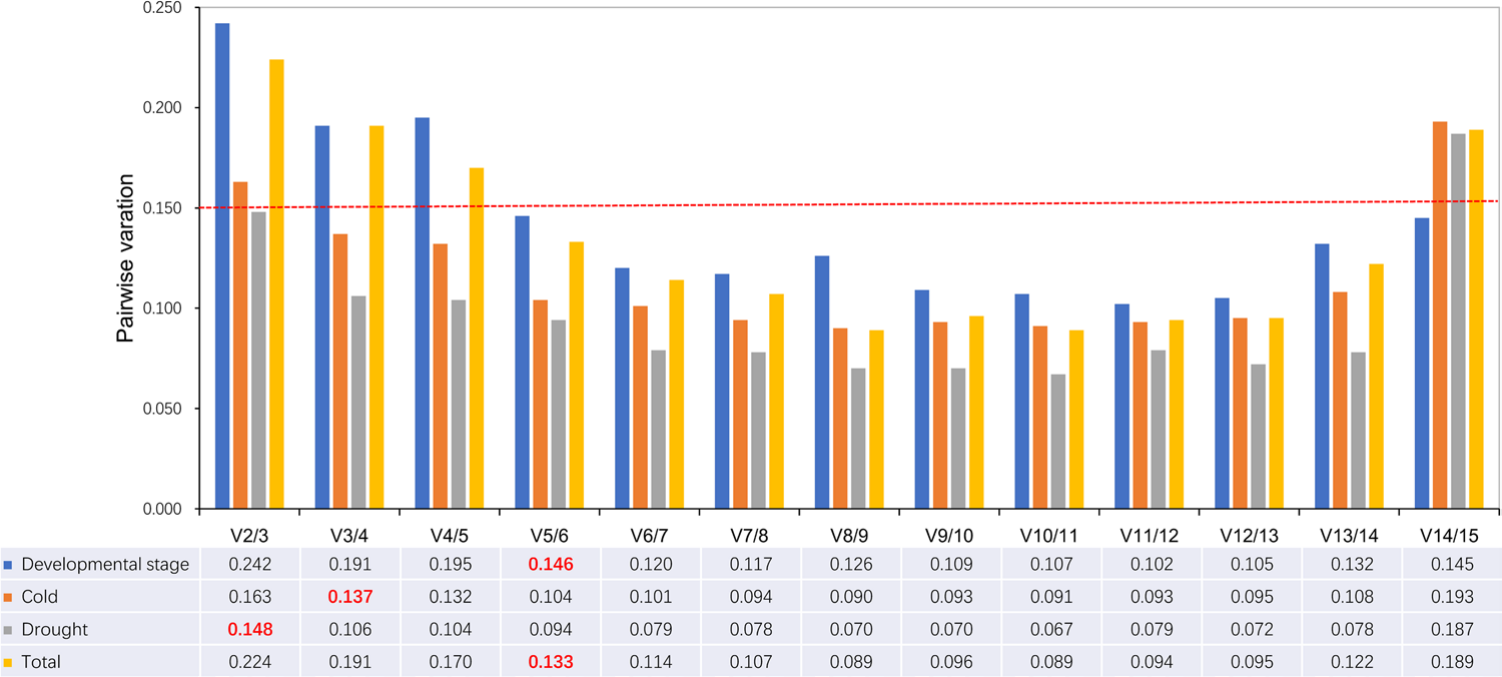
Pairwise varation to determine the optimal number of control genes for accurate normalization. The pairwise variation (V_n_/V_n + 1_) was analyzed between the NFs (NFn and NFn+1) using geNorm software, where n is the number of genes involved in the NF. Red figures indicate the optimal number of genes for normalization.

Analyzation on the pairwise variation of candidate RGs was displayed in Fig. 3. For both the total samples and samples developed at different stages, V5/6 was below 0.15, accordingly, five RGs was the optimal number. For samples subjected to cold stress treatment, V3/4 was lower than 0.15, thus 3 RGs are required, while for samples subjected to drought stress treatment, 2 RGs are needed. Results of pairwise variation was consistent with that of the stability measurements. RGs of samples subjected to cold stress or drought stress treatment demonstrated lower average pairwise variation and higher stability. In contrast, RGs of both the total samples and samples at various growth stage behaved unstable and showed higher pairwise variation.

### NormFinder analysis

NormFinder software was also applied to analyze the pairwise variation of candidate RGs. Expression stability values of genes were shown in Table 3. Genes with lower SV was considered to be stable, and suitable for RGs. Stable genes in samples at different developmental stages were *PP2A59γ, UBCE32* and *RPL5B. RPL13* and *RPL5B* were the most suitable RGs in cold stress treatment. *PP2A59γ* was the most stable RG in drought stress treatment. *PP2A59γ, RPL13*, *RPL5B*, and *UBCE32* were the most suitable RGs for total samples. However, *ACTβ* was the most unstable gene in all of the samples, which agreed with the results from geNorm analysis.

**Table 3:** The rank of RGs for normalization and their expression stability values calculated by the NormFinder program.

Rank position	Developmental stage		Cold		Drought		Total	
	Gene	Stability value	Gene	Stability value	Gene	Stability value	Gene	Stability value
1	*PP2A59γ*	0.205	*RPL13*	0.075	*PP2A59γ*	0.084	*PP2A59γ*	0.145
2	*UBCE32*	0.238	*RPL5B*	0.095	*RPL13*	0.252	*RPL13*	0.157
3	*RPL5B*	0.243	*PP2A59γ*	0.157	*UBCE32*	0.328	*UBCE32*	0.160
4	*PP2A57θ*	0.291	*EIF4A-14*	0.174	*CPY20-1*	0.334	*RPL5B*	0.179
5	*RPL13*	0.305	*UBCE32*	0.184	*UPL-RHF2A*	0.408	*CPY20-1*	0.208
6	*CPY20-1*	0.313	*CPY20-1*	0.187	*PP2A57θ*	0.443	*PP2A57θ*	0.222
7	*G6PDH*	0.313	*PP2A57θ*	0.219	*RPL5B*	0.444	*EIF4A-14*	0.236
8	*EIF4A-14*	0.352	*G6PDH*	0.268	*UBC18*	0.460	*G6PDH*	0.311
9	*TUBα3*	0.356	*EF1α*	0.281	*EIF4A-14*	0.462	*EF1α*	0.320
10	*UBC18*	0.367	*UPL-RHF2A*	0.333	*EF1α*	0.481	*UBC18*	0.385
11	*EF1α*	0.494	*TUBα3*	0.437	*ABCC8*	0.573	*TUBα3*	0.393
12	*GAPDH*	0.528	*UBC18*	0.496	*TUBα3*	0.636	*UPL-RHF2A*	0.397
13	*ABCC8*	0.634	*GAPDH*	0.521	*G6PDH*	0.689	*GAPDH*	0.512
14	*UPL-RHF2A*	0.730	*ABCC8*	0.647	*GAPDH*	0.819	*ABCC8*	0.542
15	*ACTβ*	0.903	*ACTβ*	1.276	*ACTβ*	1.669	*ACTβ*	1.167

### BestKeeper analysis

BestKeeper analysis determined stable RG according to the Ct values of each gene. Genes with high coefficient of determination (r), low SD and CV are considered stable. According to SD, CV, and r of Ct value, the expression stability of candidate RGs was ranked corresponding to the different developmental stages, cold and drought stress treatment, as well as the total samples. Then the geomean value of each RG was calculated and used to rank the fifteen RGs (Table 4). The results indicated that the optimal RG was *ABCC8* for developmental stages, *UBC18* for cold treatment, and *RPL13* for both drought treatment and the total samples. The most unstable RG was *ACTβ* except for developmental stage, in which the most unreliable RG was *UPL-RHF2A*.

**Table 4:** The rank of RGs for normalization calculated by the BestKeeper program.

Rank position	Developmental stage		Cold		Drought		Total	
	Gene	Geomean	Gene	Geomean	Gene	Geomean	Gene	Geomean
1	*ABCC8*	2.466	*UBC18*	3.302	*RPL13*	2.000	*RPL13*	2.154
2	*GAPDH*	3.634	*ABCC8*	3.557	*CPY20-1*	2.466	*CPY20-1*	3.000
3	*RPL13*	4.579	*GAPDH*	3.733	*UBCE32*	3.420	*UBCE32*	3.420
4	*CPY20-1*	4.718	*RPL5B*	3.826	*PP2A59γ*	3.826	*UBC18*	4.041
5	*G6PDH*	4.762	*PP2A59γ*	4.642	*UBC18*	4.160	*RPL5B*	4.932
6	*EIF4A-14*	4.946	*UBCE32*	5.192	*ABCC8*	6.782	*EIF4A-14*	4.946
7	*UBC18*	5.848	*EIF4A-14*	6.415	*RPL5B*	6.910	*PP2A59γ*	5.518
8	*RPL5B*	6.316	*RPL13*	7.114	*G6PDH*	8.173	*ABCC8*	6.952
9	*UBCE32*	6.542	*UPL-RHF2A*	7.343	*PP2A57θ*	8.243	*PP2A57θ*	7.319
10	*PP2A59γ*	6.604	*EF1α*	7.764	*UPL-RHF2A*	8.573	*TUBα3*	9.524
11	*EF1α*	7.958	*PP2A57θ*	8.143	*EIF4A-14*	10.288	*UPL-RHF2A*	10.260
12	*PP2A57θ*	9.655	*CPY20-1*	8.243	*EF1α*	10.298	*GAPDH*	10.537
13	*ACTβ*	12.897	*G6PDH*	10.459	*TUBα3*	10.483	*G6PDH*	11.334
14	*TUBα3*	12.974	*TUBα3*	12.083	*GAPDH*	10.801	*EF1α*	12.209
15	*UPL-RHF2A*	13.976	*ACTβ*	14.659	*ACTβ*	15.000	*ACTβ*	15.000

### Comprehensive stability analysis of reference genes

Based on the ranking results of expression stability by means of the four methods (ΔCt, geNorm, NormFinder, and BestKeeper), the geomean value of each RG was calculated and applied to determine the comprehensive rank of the fifteen RGs in developmental stage, cold treatment, drought treatment, and the total samples. Results in Table 5 showed that *ACTβ* was the most unstable RG (single low-ranked RG). The top-ranked single RG is *PP2A59γ* for developmental stage, *RPL5B* for cold treatment, and *RPL13* for both drought treatment and the total samples. According to the number of RGs suggested by geNorm and the ranking listed in Table 5, the best combination of RGs was *PP2A59γ*, *CPY20-1*, *UBCE32*, *RPL5B, and UBC18* for developmental stage, *RPL5B*, *RPL13*, and *PP2A59γ* for cold treatment, *RPL13* and *PP2A59γ* for drought treatment, and *RPL13*, *UBCE32*, *RPL5B*, *CPY20-1* and *PP2A59γ* for the total samples.

**Table 5:** The comprehensive ranking of RGs for normalization.

Rank position	Developmental stage	Cold	Drought	Total
	gene	gene	gene	gene
1	*PP2A59γ*	*RPL5B*	*RPL13*	*RPL13*
2	*CPY20-1*	*RPL13*	*PP2A59γ*	*UBCE32*
3	*UBCE32*	*PP2A59γ*	*CPY20-1*	*RPL5B*
4	*RPL5B*	*UBCE32*	*UBCE32*	*CPY20-1*
5	*UBC18*	*EIF4A-14*	*UBC18*	*PP2A59γ*
6	*G6PDH*	*UBC18*	*RPL5B*	*EIF4A-14*
7	*RPL13*	*PP2A57θ*	*UPL-RHF2A*	*PP2A57θ*
8	*ABCC8*	*CPY20-1*	*PP2A57θ*	*UBC18*
9	*PP2A57θ*	*ABCC8*	*ABCC8*	*G6PDH*
10	*GAPDH*	*G6PDH*	*EIF4A-14*	*TUBα3*
11	*EIF4A-14*	*GAPDH*	*EF1α*	*UPL-RHF2A*
12	*TUBα3*	*EF1α*	*G6PDH*	*ABCC8*
13	*EF1α*	*UPL-RHF2A*	*TUBα3*	*GAPDH*
14	*UPL-RHF2A*	*TUBα3*	*GAPDH*	*EF1α*
15	*ACTβ*	*ACTβ*	*ACTβ*	*ACTβ*

### Validation on the stability of reference genes

**Figure 4:**
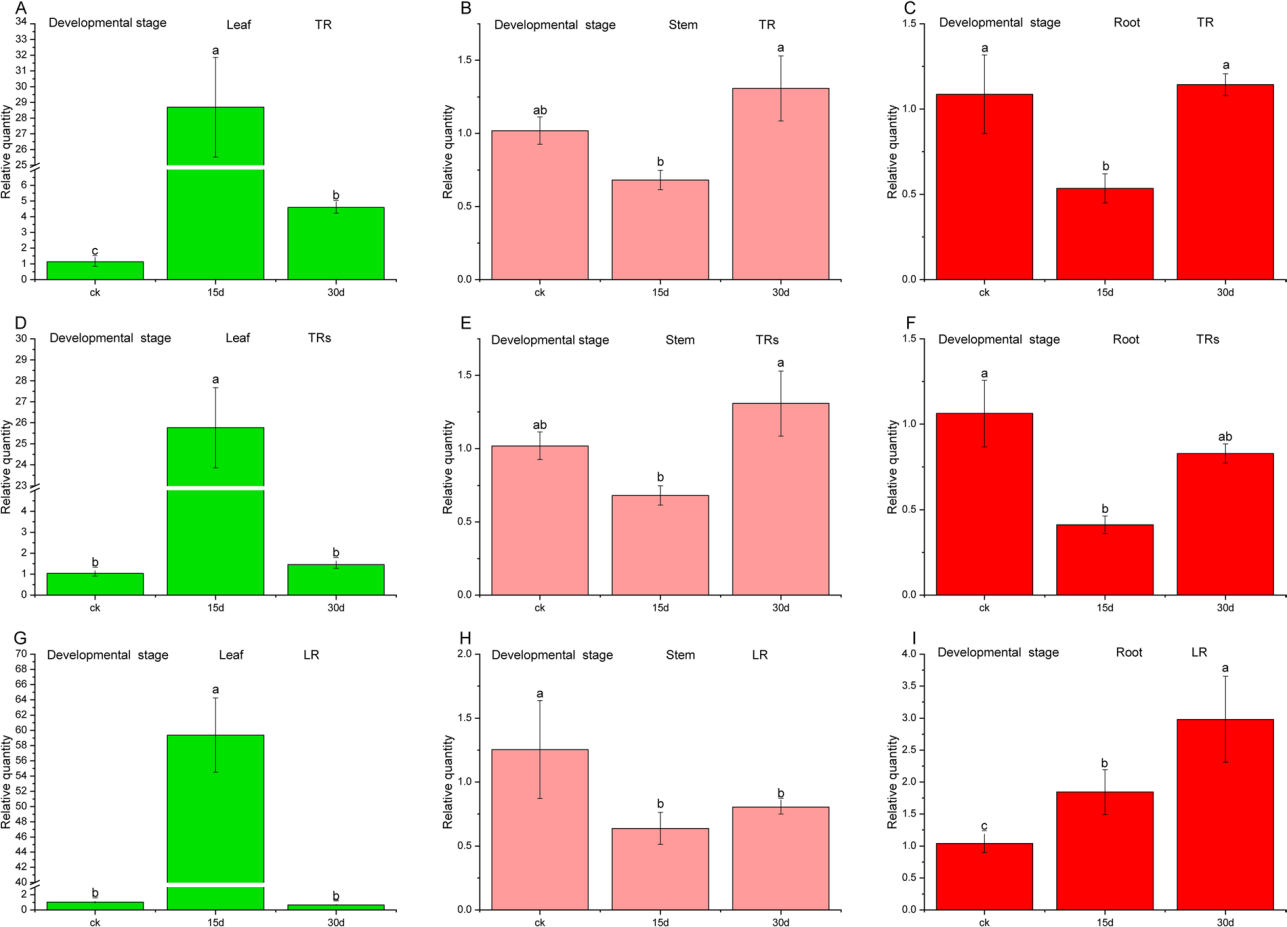
Relative quantity of *UDGPase* in different developmental stage with different RGs after normalization. Samples A, B, C were normalized with a single top-ranked RG (TR)- *PP2A59γ*. samples D, E, F were normalized with a combination of multiple top-ranked RGs (TRs)- *PP2A59γ*, *CPY20-1*, *UBCE32*, *RPL5B* and *UBC18*, while samples G, H, I were normalized with a single low-ranked RG (LR)- *ACTβ*.

**Figure 5:**
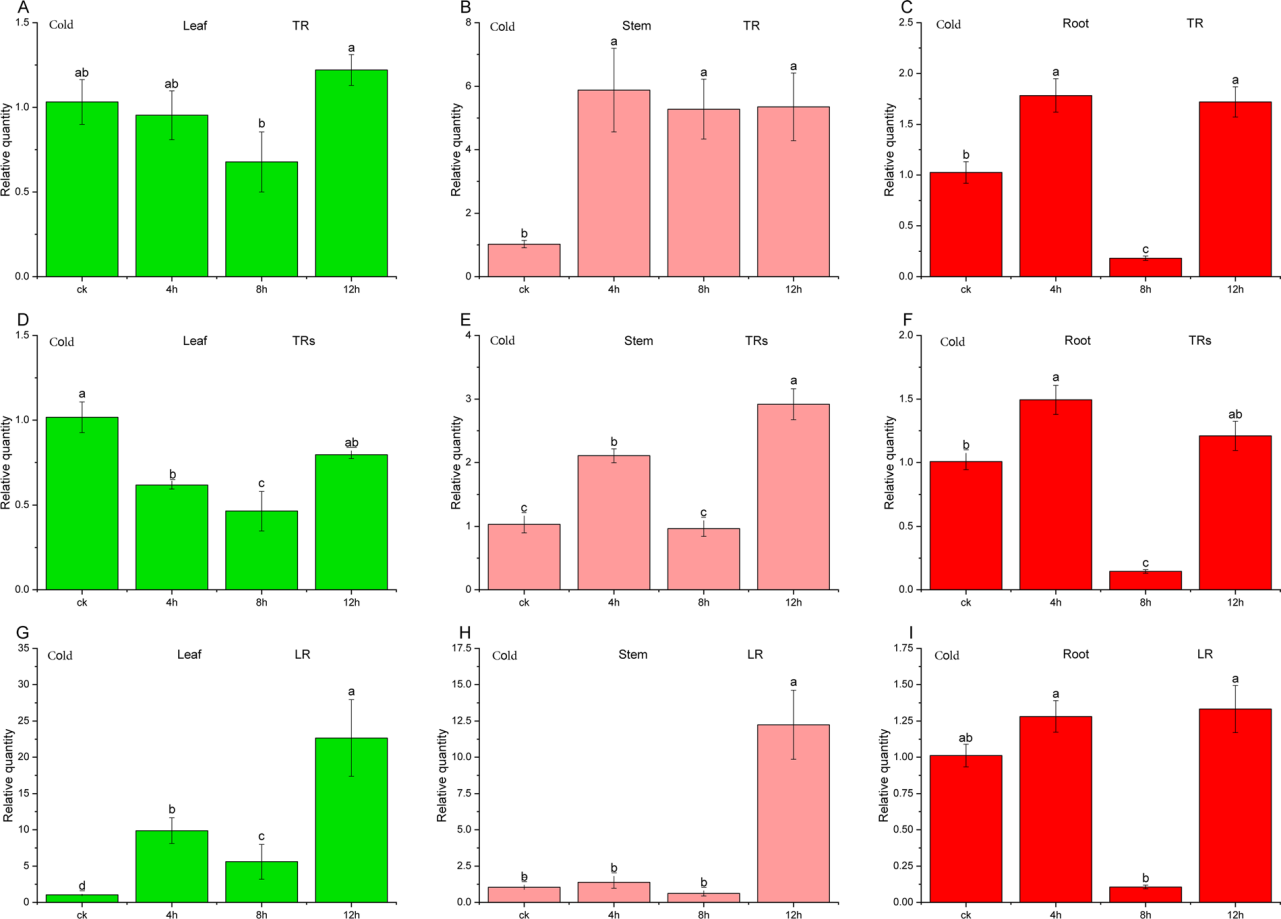
Relative quantity of *UDGPase* in cold stress with different RGs after normalization. Samples A, B, C were normalized with a single top-ranked RG (TR)- *RPL5B*. samples D, E, F were normalized with a combination of multiple top-ranked RGs (TRs)- *RPL5B*, *RPL13* and *PP2A59γ*, while samples G, H, I were normalized with a single low-ranked RG (LR)- *ACTβ*.

**Figure 6:**
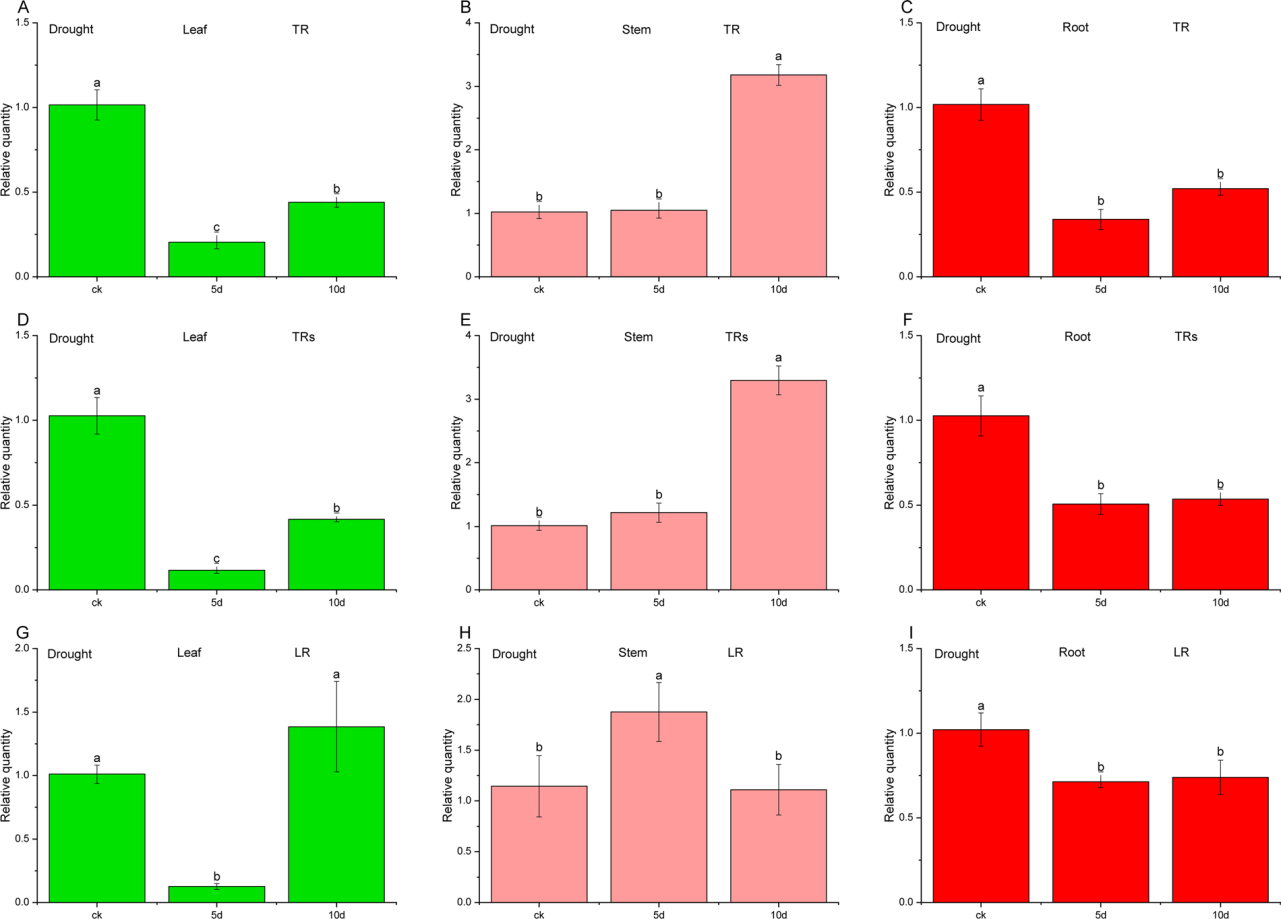
Relative quantity of *UDGPase* in drought stress with different RGs after normalization. Samples A, B, C were normalized with a single top-ranked RG (TR) - *RPL13*. samples D, E, F were normalized with a combination of multiple top-ranked RGs (TRs)- *RPL13* and *PP2A59γ*, while samples G, H, I were normalized with a single low-ranked RG (LR)- *ACTβ*.

The validity of RG or RGs, denoted as relative quality in Figs. 4 to 6, was verified by normalizing the target genes *UDGPase* (UDP glucose pyrophosphorylase) with the selected single RG, the combination of RGs and *ACTβ*. For the developmental stage of *C. pilosula*, when *PP2A59γ* alone was used for target gene expression, relative expression of *UDGPase* in leaves increased firstly and then decreased, and the gene *UDGPase* was found to be overexpressed nearly 29 times on the 15^th^ day (Fig. 4A). Relative expression of *UDGPase* in both the stems and roots decreased and then increased (Fig. 4B,C), reaching the highest on the 30^th^ day for the stems. In comparison, when normalization was performed using the *ACTβ* gene, serious deviation produced in all tissues (leaf, stem and root), and the gene *UDGPase* was found to be overexpressed nearly 58 times on the 15^th^ day (Fig. 4G–I). Using the combination of RGs including *PP2A59γ*, *CPY20-1*, *UBCE32*, *RPL5B* and *UBC18*, the expression characteristics of *UDGPase* in leaves, stems and roots was basically similar to that using *PP2A59γ* alone. However, the relative expression levels of *UDGPase* at different time points in different tissues were slightly lower than those using the most stable single RG (Fig. 4D–F).

For the cold treatment, as shown in Fig. 5, the variation characteristics of relative expression of *UDGPase* are similar in three organs whether using the most stable *RPL5B* or combination of *RPL5B*, *RPL13* and *PP2A59γ*. The relative expression levels of *UDGPase* using single stable RG were slightly higher than those using combination of RGs except for the control. Application of unstable *ACTβ* gene for normalizing target gene in root leaded to larger deviations. In the drought treatment, Both the most stable *RPL13* and combination of *RPL13* and *PP2A59γ* were the effective RGs for the relative expression of *UDGPase* of the three tissues. In contrast, using the most unstable *ACTβ* gene for normalization, although the expression of *UDGPase* in the roots was similar to that of the most stable RG or RGs, the expression bias of *UDGPase* appeared in the leaves and stems (Fig. 6).

## Discussion

Although *C. pilosula* is a traditional Chinese medicine, insufficient researches have been conducted on the internal reference genes for gene expression normalization in biosynthesis of its main components. As a result, the synthesis mechanism is not clear by now. Fortunately, Gao, J. P. *et al*. initiated the study of the internal reference gene of *C. pilosula*^8^. In their work, *GAPDH* was selected as RG to study the expression characteristics of *UDGPase* gene in polysaccharide synthesis. In our study, more stable RG and RGs combinations than *GAPDH* were found. Based on RT-qPCR study of *C. pilosula*, systematic selection and validation analysis of reliable RGs for gene expression normalization were carried out. More importantly, candidate RGs, used in the selection of stable RGs, were retrieved from the transcriptome data of *C. pilosula*, and then screened according to the CV, log2FC values of the expression level of each gene in all samples.

RT-qPCR was used to determine the expression of the candidate RGs under the conditions of cold stress, drought stress and developmental stages, and finally the stable RGs were selected. This study confirmed that RNA-seq technology was a feasible and reliable way to select RGs for *C. pilosula*. To obtain suitable RG, four calculation methods including ΔCt^38^, geNorm^39^, NormFinder^40^ and BestKeeper^41^ were applied in analyzing the stability of candidate RGs. The rankings derived from the four methods were slightly different due to the changed algorithms^45^. Taking full consideration of the ranks obtained from the four methods, the geometric means of the four rankings were calculated and used to get an integrated ranking. Accordingly, the suitable RG or the combination of RGs were selected. Surprisingly, some RGs commonly used in other plants, such as *ACTβ*, *EF1α* and *TUBα3*, are considered as unstable RGs in our study^9,11,12,19,31^, which were consistent with the analysis results of Ct values and their range of interquartile. This indicated that genes with too high or too low expression abundance were not suitable for RGs, as well as the genes with a wide interquartile range of Ct values.

Some candidate RGs that ranked at the top of the RNA-Seq analysis were not always stable in RT-qPCR test. This study also showed that the single optimal RG is *PP2A59γ* for developmental stage, *RPL5B* for cold treatment, and *RPL13* for drought treatment. Single RG can be used for gene expression analysis, as is used in most studies. However, disturbing factors such as extremely cold and drought climates during the period of growth probably leaded to deviation in gene expression by using single RG. Therefore, two or more RGs are recommended for reliable gene expression^43^. Suitable combinations of RGs were obtained as follows. *PP2A59γ*, *CPY20-1*, *UBCE32*, *RPL5B, and UBC18* were the optimal combination RGs for developmental stage, *RPL5B*, *RPL13*, and *PP2A59γ* for cold treatment, and the combination of *RPL13* and *PP2A59γ* for drought treatment. The combination of RGs could reduce the influencing of experimental factors on the gene expression, accordingly, it was more accurate and reliable than single RG in the normalization of gene expression^9,25,45^. Comprehensive analysis on the selected RGs recommended five RGs combination for gene expression of *C. pilosula*, which are *RPL13*, *UBCE32*, *RPL5B*, *CPY20-1*, and *PP2A59*.

## Materials and Methods

### Plant material

One-year-old seedlings of *C. pilosula* were collected as test plant from Tanchang, Gansu province, China on April 5^th^ 2018. The seedlings were cultivated in sandy soil at a temperature of 25 ± 2 °C, relative humidity of 55–60%, irradiance of 300 μmol m^−2^ s^−1^ and 14 h photoperiod (6:00–20:00 h). After 30 days cultivation, test plants were selected from the uniformly grown seedlings with the length of vines between 30 and 50 cm. These plants were subjected to three treatments, which are the normal watering and fertilization for developmental stages study, cold-stress treatment and drought-stress treatment. For developmental stages study, samples of leaf, stem and root were collected after 30, 45 and 60 days. Cold-stress treatment were conducted in incubator under 4 ± 2 °C for 0, 4, 8 and 12 h, respectively. The leaf, stem and root were collected correspondingly from the treated and untreated plants. Drought-stress treatment were carried out by controlling the relative water content of soil at 50 ± 5%, with the normally irrigated soil as controls. The leaf, stem and root were collected after 5 and 10 days from the drought-stress and normally irrigated plants. Three plants were sampled for each replicate and three replicates were required for each treatment. All samples were immediately frozen in liquid nitrogen, and kept frozen at −80 °C for analyses.

### RNA isolation and cDNA synthesis

Total RNA was extracted from each sample using the RNAprepPure Plant Kit DP441 (Tiangen Biotech CO., LTD, Beijing, China) according to the manufacturer’s instructions, followed by DNase I (Tiangen Biotech CO., LTD, Beijing, China) treatment to eliminate DNA contamination. The integrity of RNA was determined by 1.5% agarose gel electrophoresis. The purity and concentration of total RNA was determined using NanoDrop 2000 Spectrophotometer (Thermo Scientific, Waltham, MA, US). RNA samples with a concentration higher than 60 ng/μL and a ratio of A260/A280 between 1.8 and 2.0 were required for cDNA preparation. Synthesis of cDNA was conducted in the PrimerScript™ RT cDNA Synthesis Kit (TaKaRa Bio Inc., Dalian, China) using 1.0 μg RNA solution. The obtained cDNA was then diluted with 10 times nuclease-free water to prepare RT-qPCR.

### Selection of candidate reference genes

Transcriptome sequencing was performed on *C. pilosula* samples collected from different growth stages adopting paired-end sequencing technology on an Illumina Hi-Seq™ 4000 platform. After assembling and annotating the transcriptome data, the gene expression level (GEL) was analyzed by calculating TPM (Transcripts per kilobase of exon model per million mapped reads) with the help of EdgeR package^46^. Multiples of gene expression difference was calculated from the log fold change (FC) value. The related data has not been officially published. According to the literature^36,37^, the conventional RGs include *ACT*, *GAPDH*, *G6PDH*, *EF*, *18* *S rRNA*, *UBQ*, *TUB*, *EIF*, *UBC*, *H3*, *PGK*, *TEF*, *CYP*, and *NADHD*. The related genes were retrieved from the transcriptomic data of *C. pilosula*, and the gene expression levels including values of CV(coefficient of variation) and log FC were calculated. In general, the lower the CV value is, the more stable the expression of relevant genes becomes. Under the conditions of CV < 0.5 and |log_2_FC| < 0.2, the candidate reference RGs were screened from the genes which ranked high in stability.

### Primer design and RT-qPCR

Primers were designed using Primer5 software based on the sequences retrieved from the RNA-Seq dataset of *C. pilosula*. The criteria for primer design were as follows: primer lengths in 20–24 bp, GC content of 45–55%, melting temperature at 58 °C, and amplicon lengths in 100–250 bp. Primer information for the candidate RGs was listed in Table 2. Reactions were conducted using TB Green Premix Ex Taq II kit (Takara) and amplified on QuantStudio^TM^ 6 Flex Real-Time PCR system (ABI Technologies) in a final volume of 10 μL according to the manufacturer’s instructions. Melting curve analyses were done following the amplification cycles in order to examine the specificity of the reactions. Amplification efficiencies were calculated using the QuantStudio^TM^ Real-Time PCR software (ver 1.1). The PCR efficiency of each primer pair was determined by the slope of the standard curve according to the equation E = [10(−1/slope) − 1] × 100%^47^. Efficiency values should be between 90% and 110%.

### Data analysis

The four most commonly used methods including ΔCt^38^, geNorm^39^, NormFinder^40^ and BestKeeper^41^, were adopt to calculate and identify the expression stability of candidate reference genes. The ΔCt method displays the pairwise comparisons by calculating the standard deviation (SD) of each pair candidate RGs and the average SD of each gene. The lower the average SD is, the more stable the RG performs. The geNorm algorithm calculates the expression stability value (M value) and pairwise variation (Vn/n + 1) for all candidate genes. Lower M values suggests higher stability of gene expression. The Vn/n + 1 value determines the optimal number of RGs for accurate normalization. A cut-off value of Vn/n + 1 < 0.15 indicates that an additional RG makes no significant contribution to the normalization^42^. NormFinder program is based on the ANOVA model, which reveals expression variation by calculating the stability value (SV) when using RGs for normalization. Lower SV indicates higher stability. The BestKeeper method is an Excel-based tool using pairwise correlations, which discloses the expression level of all candidate genes by calculating the SD, coefficient of correlation (r) and coefficient of variance (CV). After rejecting the SD values of RGs higher than one, the candidate RGs was ranked according to the three indexes. The most stable gene expression exhibits the lowest CV ± SD value. Based on the results derived from the four methods, the geometric mean of each candidate gene was calculated to obtain its overall final ranking. A lower geometric mean of ranking indicates more stable expression.

### Validation of reference genes

In order to validate the selected RGs under different treatment conditions, relative expression analyses of *UDGPase* with published data were performed using the 2^−ΔΔCt^ method^17^. The effect of the single RG and the combination of RGs in normalizing the relative expression level of the target gene were compared to determine whether the combination of internal RGs can improve the reliability and accuracy of RT-qPCR results. Gene expression was also normalized using the most unstable *ACTβ* gene for comparison.

## Conclusions

This research provided the optimal single RG and combination of RGs for *C. pilosula*. The combination of *PP2A59γ*, *CPY20-1*, *UBCE32*, *RPL5B, and UBC18* are suitable for developmental stage, *RPL5B*, *RPL13*, and *PP2A59γ* for cold treatment, and in drought treatment, *RPL13* and *PP2A59γ* were the optimal combination of RGs. These combinations of RGs are recommended as reference genes in RT-qPCR analysis of *C. pilosula*. This study also demonstrated the validity of using RNA-Seq expression data for the determination of candidate reference genes for RT-qPCR analysis.

## Supplementary information

Table S1.
