# Supplementary material for: Selection and Validation of Reference Genes for Gene Expression Studies in Codonopsis pilosula Based on Transcriptome Sequence Data
Source: Sci Rep. 2020 Jan 28;10:1362. doi: 10.1038/s41598-020-58328-5 (PMC6987187; doi:10.1038/s41598-020-58328-5)
Supplement: Supplementary file 1 — Table S1. [file 41598_2020_58328_MOESM1_ESM.pdf]

Supplementary Information for

Selection and Validation of Reference Genes for Gene Expression Studies in *Codonopsis pilosula* Based on Transcriptome Sequence Data

Lijun Liang [1,2, 3], Zhigui He [4, 5], Haizheng Yu [1, 3], Erhuan Wang [6], Xiaojiang Zhang [6,] Bingxue Zhang [1, 3], Chenlu Zhang [7] and Zongsuo Liang [1, 4, 8, *]

[1]Institute of soil and water conservation, CAS & MWR, Yangling 712100, P. R. China.
[2]College of Landscape Architecture, Zhejiang Agriculture & Forestry University, Hangzhou 311300, P. R. China.
[3]University of Chinese Academy of Sciences, Beijing 100049, P. R. China.
[4]College of Life Sciences, Northwest A&F University, Yangling 712100, P. R. China.
[5]Institute of Food Science and Biological Engineering, Guilin Tourism University, Guilin 541006, P. R. China.
[6]Buchang Pharmaceuticals Co., Ltd., Xi'an 712000, P. R. China.
[7]School of Biological Science and Engineering, Shaanxi University of Technology, Hanzhong 723000, P. R. China.
[8]College of Life Sciences, Key Laboratory of Plant Secondary Metabolism and Regulation of Zhejiang Province, Zhejiang Sci-Tech University, Hangzhou 310018, P. R. China.
*Correspondence: liangzs@ms.iswc.ac.cn; Tel: +86 0571-86843684

This PDF file includes:

Table S1. The top 50 stably genes selected from *C. pilosula* transcriptome data

Table S1. The top 50 stably genes selected from C. pilosula transcriptome data

Gene symbol	Gene_ID	Length	Gene_description	ck_TPM	tr_TPM	meam_TPM	SD	CV	log2FC	Rank
ACTβ	TRINITY_DN26124_c2_g1	1715	beta-actin	17.38	17.93	17.66	0.39	0.02	0.04	1
UPL-RHF2A	TRINITY_DN21607_c0_g5	1797	E3 ubiquitin-protein ligase RHF2A	26.40	27.41	26.91	0.71	0.03	0.05	2
UBC32	TRINITY_DN18331_c0_g1	1353	Ubiquitin-conjugating enzyme E2 32	69.65	74.14	71.90	3.17	0.04	0.09	3
EF1α	TRINITY_DN18635_c0_g3	1523	Elongation factor 1-alpha	52.46	48.70	50.58	2.66	0.05	-0.11	4
PP2A59γ	TRINITY_DN18767_c2_g2	2007	serine/threonine protein phosphatase 2A 59 kDa regulatory subunit B' gamma isofor	27.29	29.73	28.51	1.73	0.06	0.12	5
RPL13	TRINITY_DN23412_c1_g10	903	60S ribosomal protein L13	108.34	99.10	103.72	6.53	0.06	-0.13	6
PP2A57θ	TRINITY_DN23680_c1_g1	2664	Serine/threonine protein phosphatase 2A 57 kDa regulatory subunit B' theta isoform	23.84	21.73	22.79	1.49	0.07	-0.13	7
RPL5B	TRINITY_DN19252_c0_g4	1754	60S ribosomal protein L5-2	153.53	139.70	146.62	9.78	0.07	-0.14	8
UBC18	TRINITY_DN23455_c0_g10	1136	ubiquitin-conjugating enzyme E2 18	55.72	61.58	58.65	4.14	0.07	0.14	9
CYP20-1	TRINITY_DN16482_c0_g3	3760	Peptidyl-prolyl cis-trans isomerase CYP20-1	48.65	54.17	51.41	3.90	0.08	0.16	10
G6PDH	TRINITY_DN22563_c0_g2	3910	Glucose-6-phosphate 1-dehydrogenase 4, chloroplastic	5.37	6.06	5.72	0.49	0.09	0.17	11
EIF4A14	TRINITY_DN19408_c0_g2	2466	Eukaryotic initiation factor 4A-14	73.99	84.05	79.02	7.11	0.09	0.18	12
ABCC8	TRINITY_DN20730_c0_g5	2513	ABC transporter C family member	5.37	6.15	5.76	0.55	0.10	0.20	13
GAPDH	TRINITY_DN16710_c1_g1	1856	NADP-dependent glyceraldehyde-3-phosphate dehydrogenase	59.82	68.51	64.17	6.14	0.10	0.20	14
TUBα3	TRINITY_DN16768_c1_g2	2401	Tubulin alpha-3 chain	98.40	112.74	105.57	10.14	0.10	0.20	15
TUBβ2	TRINITY_DN14966_c4_g4	1612	Tubulin beta-2 chain	297.37	351.91	324.64	38.57	0.12	0.24	16
RPL5B	TRINITY_DN19520_c0_g1	1330	60S ribosomal protein L5-2	229.64	189.88	209.76	28.11	0.13	-0.27	17
UPL-RNF170	TRINITY_DN19225_c3_g2	2002	E3 ubiquitin-protein ligase RNF170	8.56	10.48	9.52	1.36	0.14	0.29	18
AP1	TRINITY_DN17466_c0_g1	1900	AP-1 complex subunit sigma-2	21.23	26.07	23.65	3.42	0.14	0.30	19
UBC1	TRINITY_DN23759_c0_g1	1869	Ubiquitin-conjugating enzyme E2 1	254.30	312.84	283.57	41.39	0.15	0.30	20
EF1α	TRINITY_DN20615_c1_g2	2038	Elongation factor 1-alpha	1461.19	1826.83	1644.01	258.55	0.16	0.32	21
UPL-COP1	TRINITY_DN23695_c1_g2	2017	E3 ubiquitin-protein ligase COP1	17.60	22.10	19.85	3.18	0.16	0.33	22
UBC24	TRINITY_DN16764_c0_g2	4634	ubiquitin-conjugating enzyme E2 24	47.95	60.28	54.12	8.72	0.16	0.33	23
eiF4A3	TRINITY_DN23276_c0_g1	1774	Eukaryotic initiation factor 4A-3	103.35	131.37	117.36	19.81	0.17	0.35	24
UPL-SINAT3	TRINITY_DN15783_c0_g1	1866	E3 ubiquitin-protein ligase SINAT3	12.78	16.28	14.53	2.47	0.17	0.35	25
TUBβ1	TRINITY_DN20462_c0_g10	2352	Tubulin beta-1 chain	65.40	51.33	58.37	9.95	0.17	-0.35	26
TUBα3	TRINITY_DN15107_c1_g2	1919	Tubulin alpha-3 chain	902.05	704.43	803.24	139.74	0.17	-0.36	27
G6PDH	TRINITY_DN18566_c0_g2	2940	Glucose-6-phosphate 1-dehydrogenase, cytoplasmic isoform	256.91	200.48	228.70	39.90	0.17	-0.36	28
UBC36	TRINITY_DN23137_c1_g2	3393	Ubiquitin-conjugating enzyme E2 36	299.47	385.93	342.70	61.14	0.18	0.37	29
UBC2	TRINITY_DN17079_c1_g1	1062	Ubiquitin-conjugating enzyme E2 2	55.02	71.92	63.47	11.95	0.19	0.39	30
CYP40	TRINITY_DN22390_c1_g2	1810	Peptidyl-prolyl cis-trans isomerase CYP40	21.78	16.52	19.15	3.72	0.19	-0.40	31
TUBγ1	TRINITY_DN16040_c0_g6	2194	Tubulin gamma-1 chain	11.45	15.18	13.32	2.64	0.20	0.41	32
AP4	TRINITY_DN16652_c0_g3	1294	AP-4 complex subunit sigma	35.13	46.58	40.86	8.10	0.20	0.41	33
RPL35A	TRINITY_DN21806_c0_g4	2668	60S ribosomal protein L35a-1	15.17	20.37	17.77	3.68	0.21	0.43	34
AP3	TRINITY_DN17237_c2_g2	1642	AP-3 complex subunit sigma	14.76	10.89	12.83	2.74	0.21	-0.44	35
PP2A57ι	TRINITY_DN18527_c0_g4	4699	Serine/threonine protein phosphatase 2A 57 kDa regulatory subunit B' iota isoform	8.95	6.60	7.78	1.66	0.21	-0.44	36
UBC7	TRINITY_DN15567_c0_g2	1593	Ubiquitin-conjugating enzyme E2 7	143.51	194.62	169.07	36.14	0.21	0.44	37
TUBα3	TRINITY_DN16768_c1_g7	1731	Tubulin alpha-3 chain	9.40	12.97	11.19	2.52	0.23	0.46	38
UPL-RMA1	TRINITY_DN16041_c1_g9	2677	E3 ubiquitin-protein ligase RMA1H1	91.25	126.69	108.97	25.06	0.23	0.47	39
UBC17	TRINITY_DN25895_c2_g1	1932	Ubiquitin-conjugating enzyme E2-17	454.63	633.98	544.31	126.82	0.23	0.48	40
CYP18-2	TRINITY_DN24338_c0_g2	1835	Peptidyl-prolyl cis-trans isomerase CYP18-2	7.28	10.28	8.78	2.12	0.24	0.50	41
AP2	TRINITY_DN18795_c1_g1	1973	AP-2 complex subunit sigma	32.01	46.38	39.20	10.16	0.26	0.53	42
PP2A	TRINITY_DN17724_c1_g2	2618	Serine/threonine-protein phosphatase PP2A catalytic subunit	59.19	86.52	72.86	19.33	0.27	0.55	43
UBC25	TRINITY_DN23093_c1_g4	2611	ubiquitin-conjugating enzyme E2 25	13.11	19.72	16.42	4.67	0.28	0.59	44
PP2A55β	TRINITY_DN21666_c1_g5	3583	Serine/threonine protein phosphatase 2A 55 kDa regulatory subunit B beta isoform	34.26	52.02	43.14	12.56	0.29	0.60	45
CYP21-1	TRINITY_DN16353_c0_g1	1212	Peptidyl-prolyl cis-trans isomerase CYP21-1	12.87	20.29	16.58	5.25	0.32	0.66	46
ABCG24	TRINITY_DN20277_c0_g3	1128	ABC transporter G family member	5.24	9.25	7.25	2.84	0.39	0.82	47
GAPDH	TRINITY_DN24417_c0_g1	2076	Glyceraldehyde-3-phosphate dehydrogenase, chloroplastic	182.40	324.78	253.59	100.68	0.40	0.83	48
AP3M	TRINITY_DN16755_c0_g6	1648	AP-3 complex subunit mu	5.84	10.56	8.20	3.34	0.41	0.85	49
UBC23	TRINITY_DN23426_c1_g5	3537	ubiquitin-conjugating enzyme E2 23	7.56	14.22	10.89	4.71	0.43	0.91	50
